# Genitourinary symptoms in women with breast cancer: frequency, severity and impact

**DOI:** 10.1007/s00520-025-09297-w

**Published:** 2025-03-10

**Authors:** Antonia Pearson, Haryana M. Dhillon, Jill Chen, Rachel Campbell, Janine Lombard, Martha Hickey, Belinda E. Kiely

**Affiliations:** 1 Sydney Medical School, University of Sydney, Camperdown, NSW Australia; 2 Medical Oncology, Northern Beaches Hospital, Frenchs Forest, NSW Australia; 3 Psycho-Oncology Cooperative Research Group, School of Psychology, Faculty of Science, The University of Sydney, Camperdown, NSW Australia; 4 Newcastle Private Hospital, Newcastle, NSW Australia; 5 Medical Oncology, Calvary Mater Newcastle, Newcastle, NSW Australia; 6 University of Newcastle, Newcastle, NSW Australia; 7 University of Melbourne, Melbourne, VIC Australia; 8 Department of Obstetrics, Gynaecology and Newborn Health, University of Melbourne and the Royal Women’S Hospital, Melbourne, VIC Australia; 9 Trials Centre, NHMRC Clinical, University of Sydney, Camperdown, NSW Australia; 10 Macarthur Cancer Therapy Centre, Campbelltown Hospital, Campbelltown, NSW Australia; 11 Concord Cancer Centre, Concord Repatriation General Hospital, Concord, NSW Australia

**Keywords:** Breast cancer, Genitourinary syndrome of menopause, Vaginal atrophy, Vaginal estrogen

## Abstract

**Purpose:**

To improve understanding of genitourinary symptoms (GUS) in women with breast cancer (BC).

**Methods:**

Women with BC completed a survey assessing the type, severity, and impact of GUS experienced, and perceptions of treatment options.

**Results:**

Surveys were completed by 506 women: median age 60 years (range 30 – 83). The majority reported: being sexually active (52%); currently taking endocrine therapy (58%); and having early-stage BC (84%). 69% had GUS, with some changing (5%) or stopping (4%) endocrine therapy as a result. Vaginal dryness was the most common symptom (62%), followed by pain during penetration (41%) and itch (33%). Only 44% recalled being warned by their cancer doctor that BC treatment can cause GUS, and 38% reported never being asked about GUS. Being uncomfortable talking to a male healthcare professional was a moderate or major barrier to accessing advice and treatment for GUS in 28% of respondents. A minority reported using vaginal: lubricants (40%); moisturisers (25%); or oestrogens (16%). Amongst those using vaginal oestrogens, 45% found they helped “quite a bit” or “very much”. The most frequently reported moderate to major barrier to using vaginal oestrogens was product information warning against use in women with BC.

**Conclusions:**

Although GUS are very common in women with BC, the majority of women in our study do not recall being warned or asked about these symptoms. Healthcare professionals should initiate conversations about GUS and treatment options with women with BC to help reduce the impact of these symptoms.

## Introduction

Genitourinary Syndrome of Menopause or vulvovaginal atrophy is commonly reported in women with a history of breast cancer, with up to 75% of breast cancer survivors reporting at least one genitourinary symptom [1]. This constellation of symptoms includes: vaginal dryness; vaginal itch or burning; dysuria; urinary urgency; urinary incontinence; dyspareunia; loss of libido; and dysfunction of arousal and orgasm [2, 3].

Genitourinary symptoms (GUS) can be caused or exacerbated by breast cancer treatment. Chemotherapy-induced premature menopause, ovarian function suppression and aromatase inhibitors are most implicated [2, 4]. Chronic oestrogen depletion changes the female genital tract through reduced blood flow and secretions, increased pH, thinning of the epithelium, and loss of elasticity [2]. GUS can result in poor adherence and premature cessation of endocrine therapy [5] and can negatively impact health-related quality of life and sexual function [6].

Several treatment options are available for GUS including non-hormone based vaginal moisturisers and lubricants, oral and transdermal systemic menopausal hormone therapy, vaginal oestrogens, and vaginal laser [7]. For women with a history of breast cancer, particularly hormone driven cancers, systemic hormone therapy is not advised and vaginal oestrogen-based treatments are often avoided due to concerns that such treatments may stimulate cancer growth, increasing breast cancer recurrences [2]. However existing evidence suggests vaginal oestrogens are probably safe in women with breast cancer, with studies showing no increased risk of de novo breast cancer [8–11] or breast cancer recurrence [11–14]. International guidelines and position statements from various groups support use of vaginal oestrogens for GUS in women with breast cancer, when non-hormonal interventions (e.g., moisturisers and/or lubricants) are ineffective [15–18]. Despite this, vaginal oestrogen product labelling continues to include a warning against use in women with breast cancer; consequently many women avoid their use [19].

Medical professionals who care for women with breast cancer are frequently required to manage GUS despite most having little or no training in this. A cross-sectional survey of medical professionals involved in the care of women with breast cancer in Australia and New Zealand found that despite GUS being recognised as common in this population only 16% felt confident managing these symptoms [20].

The aim of this study was to determine the experiences of GUS in women with breast cancer. Better understanding of patients’ experience will help inform future approaches to patient care, development of treatment guidelines, and implementation of medical recommendations.

## Methods

### Study design

We conducted a cross-sectional survey. Ethics approval was granted for all sites by the Northern Sydney Local Health District Human Research Ethics Committee, 2020/ETH01955.

### Participants

Women with breast cancer of any stage and type were eligible to participate. The survey was distributed to oncology clinic attendees at 11 cancer centres in New South Wales, Australia, as well as members of the Breast Cancer Network of Australia (BCNA), Australia’s leading breast cancer consumer organisation.

### Survey instrument

A survey, in paper and online formats, was developed with input from the consumer advisory panel of Breast Cancer Trials, Australia’s leading cooperative breast cancer clinical trials group. The survey elicited respondent experiences of GUS and perceptions of treatment options.

The survey included questions about the impact of GUS on daily function, potential barriers to accessing treatment and experience discussing GUS with healthcare professionals. Symptom severity was rated as none, mild, moderate or severe for each of the following: vaginal dryness, vaginal itch, vaginal irritation, vaginal pain and pain during penetration. The following standardised validated questionnaires were included: Day-to-Day Impact of Vaginal Ageing (DIVA) [21]; and, International Consultation on Incontinence Questionnaire Female Lower Urinary Tract Symptoms (ICIQ FLUTS) [22]. The DIVA assesses quality of life across four domains: activities of daily living, emotional well-being, sexual functioning, and self-concept and body image [21]. Total scores for each domain are calculated by averaging item scores per domain. Scores range 0–4, with higher scores demonstrating greater impact of vaginal symptoms. ICIQ FLUTS is designed to evaluate female lower urinary tract symptoms including domains on filling, voiding and incontinence [22]. Total scores for each domain are calculated by summing item scores per domain. Domain scores are then added to achieve a total score out of 48 with higher scores indicating greater impact of symptoms. Demographic information was collected for each respondent, including age, education, sexual orientation, smoking history, self-reported menopausal status (at the time of survey completion), and details of breast cancer diagnosis and treatment.

Oncology clinic attendees were informed of the study during a scheduled follow-up consultation, and willing participants completed the paper survey. BCNA members were emailed a participant information sheet explaining the study with a link to the online version of the survey. Submission of a completed survey was accepted as an indication of consent.

### Analysis

Study data were collected and managed using REDCap electronic data capture tools hosted at the University of Sydney [23, 24]. Descriptive statistics were used to report frequencies, means, and medians. Chi-squared tests were used to compare symptom severity in different groups and unpaired t tests and effect sizes were used to compare DIVA and ICIQ FLUTS scores in women with no or mild symptoms to those who had moderate or severe symptoms. We compared scores for the DIVA and ICIQ FLUTS questionnaires for women reporting at least one moderate or severe GUS to those who reported none or only mild GUS.

## Results

Surveys were returned by 506 respondents: 378 from oncology clinics and 128 from BCNA.

The median age of respondents was 60 years (range 30 to 83 years). 69% reported being married or in a relationship, and 2% identified as lesbian, gay, bisexual, transgender, queer or intersex (LGBTQI +). At the time of survey completion, 52% of respondents reported being sexually active. Most respondents reported having early-stage breast cancer (84%), with 15% having metastatic cancer, and 1% unsure. The majority (88%) reported being postmenopausal. Table 1 summarises respondent characteristics.

**Table 1:** Respondent characteristics

Characteristic	*n* (%) total = 506
Menopausal status (at time of survey) Postmenopausal Perimenopausal Premenopausal Unanswered	440 (88) 42 (9) 17 (3) 7
Sexually active Yes No Prefer not to say Unanswered	261 (52) 208 (41) 33 (7) 4
Sexual Orientation Heterosexual Lesbian, gay or homosexual Bisexual Queer Different identity Prefer not to say	483 (97) 4 (< 1) 3 (< 1) 1 (< 1) 1 (< 1) 14 (3)
Breast Cancer stage Early Metastatic Unsure Unanswered	419 (84) 74 (15) 7 (1) 6
Currently on endocrine therapy Yes No Unsure Unanswered	291 (58) 202 (41) 5 (1) 8
Endocrine treatment type Aromatase inhibitor Tamoxifen Dual endocrine therapy* Fulvestrant Unsure / not reported	185 (64) 71 (24) 19 (7) 7 (2) 9 (3)

^*^ Dual endocrine therapy is defined as those on goserelin + tamoxifen or goserelin + aromatase inhibitor

Most (58%, 291/498) respondents reported currently taking endocrine therapy, including: aromatase inhibitors (185/291, 64%); selective oestrogen receptor modulators (71/291, 24%); selective oestrogen receptor degraders (7/291, 2%); and dual endocrine blockade with a gonadotropin hormone-releasing hormone (GnRH) agonist (19/291, 7%). 9% of our respondents reported changing (5%) or stopping (4%) endocrine therapy due to GUS.

### Patient-reported symptoms

**Fig. 1:**
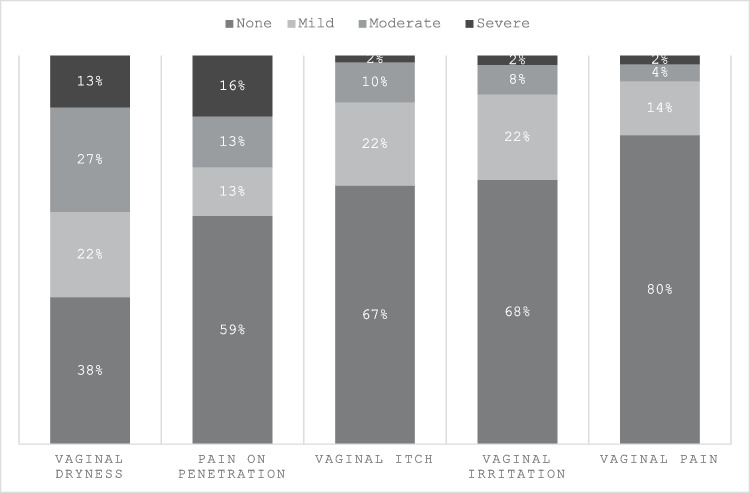
Frequency and severity of genitourinary symptoms in the last 4 weeks

At least one GUS was reported as being experienced in the last four weeks by 69% (351/506) of respondents and at least one GUS reported as moderate or severe in 47% (239/506). Vaginal dryness was the most common symptom reported (62%, 304/486), followed by pain on penetration (41%, 188/454), and itch (33%, 160/479). Of the 304 respondents with vaginal dryness in the preceding 4 weeks, 64% (195/304) rated their symptoms as moderate or severe. Similarly pain on penetration was rated moderate or severe by 70% (131/188) of respondents. Frequency and severity of GUS is summarised in Fig. 1.

When asked about their most bothersome symptom, the most frequent response was vaginal dryness (42%, 132/315), followed by dyspareunia (31%, 96/315), itch (16%, 51/315), irritation (9%, 27/315), and pain (3%, 9/315).

Vaginal dryness was more commonly reported as moderate or severe in postmenopausal women (41%) than pre/perimenopausal women (26%) (X^2^, [1, *N* = 485], *p* = *0.03).* Similarly, pain on penetration was more commonly reported as moderate or severe in post-menopausal women (28%) than pre/peri-menopausal women (14%) (X^2^, [1, *N* = 486], = 5.18, *p* = *0.02*). There was no difference in the reported frequency of moderate or severe symptoms in women currently taking endocrine therapy vs those not taking endocrine therapy (vaginal dryness X^2^ [1, *N* = 483] = 0.19, *p* = *0.66*; pain on penetration X^2^[1, *N* = 483] ≤ 0.01, *p* = *0.98*).

### Care providers

Of the 291 respondents who reported currently taking endocrine therapy, 44% (127) recalled being warned by their cancer doctor that GUS can be a side effect of BC treatment. After initiating endocrine treatment 38% (111) reported “never” being asked about GUS by their doctor, 21% (60) reported being asked about GUS only “rarely”, while 8% (22) reported “always” being asked about these symptoms.

55% (270/493) of respondents reported a preference to speak to a female healthcare professional about GUS. 43% (211/497) of respondents had spoken to a doctor about their GUS and 20% (101/497) had spoken to another healthcare professional. 31% (155/497) of respondents sought information regarding GUS elsewhere; the most common resources accessed were Google (76%, 117/155), breast cancer organisations (50%, 78/155), online forums (16%, 24/155), and Facebook (12%, 18/155).

### Impact of symptoms

Table 2 shows DIVA and ICIQ FLUTS mean scores across all domains. DIVA scores were highest (worst) for the sexual functioning and self-concept and body image domains with approximately 30% of respondents reporting their vaginal symptoms were having quite a bit or an extreme impact. Each domain DIVA score was significantly higher (p < 0.0001, Table 2) in women reporting at least one moderate to severe GUS than in women with no or only mild GUS. The self-concept and body image domain was the most impacted with the largest mean difference and effect size between groups (moderate or severe GUS compared to those with low GUS burden).

**Table 2:** DIVA quality of life questionnaire and ICIQ FLUTS questionnaire overall score and domains according to severity of symptoms

	All respondents Mean ± SD	No GUS or only mild symptoms Mean ± SD	At least one moderate or severe GUS Mean ± SD	P value***	Mean difference	CI for difference	Effect Size****
A) DIVA*	n = 486	n = 252	n = 234				
Daily activities domain	0.30 ± 0.60	0.14 ± 0.42	0.48 ± 0.72	** <** 0.0001	0.34	0.24,0.44	0.58
Emotional well-being domain	0.51 ± 0.82	0.14 ± 0.41	0.91 ± 0.95	** < **0.0001	0.77	0.64,0.90	1.06
Sexual functioning domain	1.41 ± 1.40	0.62 ± 0.99	2.24 ± 1.29	** < **0.0001	1.62	1.42,1.82	1.41
Self-concept and body image domain	1.46 ± 1.44	0.63 ± 1.01	2.30 ± 1.31	** < **0.0001	1.67	1.46,1.88	1.43
B) ICIQ FLUTS**	n = 485	n = 252	n = 233				
ICIQ FLUTS total score	8.57 ± 5.35	7.92 ± 4.91	9.27 ± 5.72	0.01	1.35	0.40,2.30	0.25
Filling domain	3.35 ± 2.11	3.19 ± 2.08	3.53 ± 2.14	0.08	0.34	−0.04, 0.72	0.16
Voiding domain	1.47 ± 1.80	1.34 ± 1.68	1.61 ± 1.92	0.09	0.27	−0.05, 0.59	0.15
Incontinence domain	3.75 ± 3.22	3.41 ± 2.90	4.13 ± 3.51	** <** 0.01	0.72	0.15, 1.29	0.22

SD standard deviation, CI confidence interval

^*^DIVA domain score out of 4. Higher scores denote worse impact of symptoms

^**^FLUTS score total out of 48: filling domain out of 16; voiding domain out of 12, incontinence domain out of 20. Higher scores denote worse impact of symptoms

^***^Independent* t* test, 0.05 significant value

^***^ Hedges’ g effect size. Small effect = 0.2, medium effect = 0.5, large effect > 0.8

The ICIQ FLUTS demonstrated the majority of women had no significant problems with urinary flow, voiding or incontinence. The most frequently reported symptoms were leakage of urine at least once per day (58/485, 12%) and nocturia at least 3 times per night (62/485, 13%). Similar to the DIVA scores, the total ICIQ FLUTS score was significantly higher (worse) in women reporting at least one moderate or severe GUS compared to those with no or mild symptoms (p = 0.01). A significant difference was seen between groups in the incontinence domain (p < 0.01; capturing leaking before toileting, during activity, for no obvious reason, when asleep, and frequency of leaking), however there were no differences in the filling and voiding domains.

### Treatment options

All respondents were asked their understanding of the safety of a range of treatment options for GUS. The proportions of respondents reporting that each treatment was “probably safe” or “very safe” was: 61% (297/486) for vaginal lubricants; 54% (262/487) for vaginal moisturisers; 21% (101/483) for vaginal oestrogens and 19% (91/480) for vaginal laser. 19% (93/483) of respondents believed vaginal oestrogens were “unsafe”.

Vaginal lubricants (40%, 197/484) and moisturisers (25%, 122/478) were the most frequently reported treatments used for GUS, followed by vaginal oestrogens (16%, 78/498), vaginal laser (4%, 19/468), vaginal dilators (3%, 14/469), and other topical treatments such as vitamin E and oils (8%, 30/385). Table 3 shows the treatments used by respondents and how much each treatment helped.

**Table 3:** Frequency of treatments used for genitourinary symptoms and reported symptom relief

Vaginal Treatment	Use of this treatment n (%)	This treatment was “somewhat” or “a lot” helpful n (%)	This treatment was “probably” or “very” safe n (%)
Lubricant	197/484 (41)	120/197 (61)	297/486 (61)
Moisturiser	122/478 (26)	61/122 (50)	262/487 (54)
Oestrogen	78/498 (16)	41/66 (62)	101/483 (21)
Laser	19/468 (4)	8/19 (42)	91/480 (19)
Dilator	14/469 (3)	5/14 (36)	117/480 (24)

For the 78 women reporting use of vaginal oestrogens, 49% (38) reported using this treatment for more than 12 months and 45% (35) found it helped their GUS “quite a bit” or “very much”. Vaginal oestrogens were most often prescribed by a general practitioner (45%, 35/78) or medical oncologist (29%, 23/78).

### Barriers to accessing treatment

Respondents were asked about barriers to discussing GUS and treatment options with healthcare professionals. The most frequently reported moderate or major barrier was discomfort talking to a male healthcare professional (28%, 127/461), followed by concerns regarding a lack of treatment options (17%, 78/459), and embarrassment (16%, 73/461).

The most frequently reported moderate to major barriers to using vaginal oestrogens were: product information warning “not to use if you have been diagnosed with breast cancer” (63%, 278/439); “my cancer doctor has not recommended vaginal oestrogens” (48%, 224/471); "worry that vaginal oestrogen will increase my risk of breast cancer returning” (58%, 269/462); and “worry that vaginal oestrogen is not safe in women diagnosed with breast cancer” (58%, 269/462).

## Discussion

Our study highlights how common GUS are for women after breast cancer, with most (69%) reporting at least one GUS in the preceding four weeks and 47% (239/506) reporting at least one moderate to severe symptom. Vaginal dryness was the most frequent and most bothersome symptom while pain on penetration was most commonly rated as severe. Despite the frequency of GUS, 59% of respondents reported “never” or “rarely” being asked about these symptoms at follow-up visits with their cancer doctor. At their worst, GUS can lead to some women prematurely ceasing endocrine therapy, with 9% of our respondents reporting changing (5%) or stopping (4%) endocrine therapy due to GUS.

Our results are consistent with previous international studies [5, 25, 26]. For example, Chin et al. reported a similar proportion of women reporting at least one GUS (63%) in their survey of 251 postmenopausal women receiving endocrine therapy for early or metastatic breast cancer [5]. In contrast GUS is reported less frequently in women without cancer, for example in a recent meta-analysis including 482,067 post-menopausal women, 45% reported experiencing GUS [27].

The finding that women are often not asked about GUS is in keeping with our previous study of 144 breast cancer healthcare professionals in Australia and New Zealand where only 55% reported regularly asking patients with breast cancer about these symptoms [20]. We also found only 44% of respondents recalled being warned by their cancer doctors that GUS can be a side effect of breast cancer treatment. Understanding this could help women subsequently to discuss their symptoms and treatment options with their cancer doctors.

Most respondents felt comfortable talking to healthcare professionals about GUS but there was a preference for females (55% of respondents), and 28% reported discomfort talking to a male healthcare professional about their symptoms. Some of these concerns could be helped by improving the way healthcare professionals communicate with patients about GUS. Encouraging doctors to initiate conversations, normalise symptoms, and offer treatments would help those patients afraid or embarrassed to mention their symptoms. The use of brief validated patient reported outcomes in clinical practice helps to facilitate discussion of sensitive topics [28], and could be implemented for GUS by asking women on endocrine therapy to complete brief symptom questionnaires prior to their oncology follow up consultations. Nearly one third of respondents had looked elsewhere for information on managing their symptoms, with Google the most used resource. Improving access to reliable sources of information is crucial to ensure patients are well informed. It is important for health professionals to both ask about GUS and direct women to reliable information.

The quality-of-life domains most impacted by vaginal symptoms were sexual functioning, and self-concept and body image with 30% of respondents reporting quite a bit or an extreme impact on sexual functioning. We also showed a significant difference in quality of life on the DIVA questionnaire between those who had moderate or severe GUS and those with no or mild GUS. Quality of life was worse in all four DIVA domains demonstrating the negative impact GUS can have on women’s daily functioning, well-being, sexual functioning, and self-esteem. This is consistent with other studies in postmenopausal women which similarly showed impairment in all domains, with sexual functioning, self-concept and body image domains being most affected [29, 30]. Reassuringly, moderate to severe urinary symptoms were infrequent.

In accordance with international guidelines [15–18], we found vaginal lubricants and moisturisers the most frequently trialled treatment for GUS (40% and 25% respectively). However, given there are no safety concerns with these non-hormone-based products, and they are generally accessible and cheap, it is surprising they are not used by more women. It was also surprising that a large proportion of women did not agree that non-hormone based treatments were probably or very safe (39% for lubricants and 46% for moisturisers). Usage can be encouraged with better patient resources on treatment of GUS and educating healthcare professionals about available treatment options. Given that one randomised trial showed vaginal moisturisers and gels were equivalent to vaginal oestrogen tablets in reducing vulvovaginal symptoms in post-menopausal women, encouraging women to use vaginal moisturisers regularly may be a simple and safe way to improve GUS in women with breast cancer [31]. Only 16% of respondents had trialled vaginal oestrogens despite international guideline recommendations for use when lubricants and moisturisers fail to ameliorate symptoms [15–18]. The reasons for such low use of vaginal oestrogens are likely multifactorial: safety concerns, low rates of prescribing, communication issues between women and healthcare providers, and lack of education of both patients and healthcare professionals involved in the management of women with breast cancer. Our previous study of healthcare professionals’ perceptions revealed only 21% frequently prescribed vaginal oestrogens with a lack of confidence and concerns regarding safety being the most reported barriers to prescribing [20].

Complicating the low prescribing rates of vaginal oestrogens is the persistence of alarming product labelling in most countries, including Australia and New Zealand, which warns against use in women with breast cancer. In our study 63% of respondents found these types of product warnings a major barrier to using vaginal oestrogens. Unfortunately, this labelling undermines confidence of both healthcare providers and patients in prescribing and using vaginal oestrogens [32], and to date efforts to remove this labelling have been unsuccessful.

In our study only 21% of respondents felt vaginal oestrogens were safe, with 19% believing them to be “unsafe”. A series of large cohort studies in women unaffected by breast cancer, including the Collaborative Group on Hormonal Factors in Breast Cancer, Women’s Health Initiative Observational Study, and Nurses’ Health Study, [8–10] have not demonstrated any association between breast cancer incidence and use of vaginal oestrogens. In women with a history of breast cancer, the limited available evidence does not show an increased risk of breast cancer recurrence or death with vaginal oestrogen use [11–13]. Other studies have shown vaginal oestrogens have minimal or no impact on serum oestrogen levels suggesting negligible systemic absorption [33–35]. A recent Danish observational study of women with early breast cancer [14] reported no increased risk of recurrence or mortality associated with vaginal oestrogen use; however an increased risk of breast cancer recurrence was seen in a subgroup analysis of women on aromatase inhibitors. This study has a number of limitations: only the subgroup analysis showed an impact on recurrence and it was not powered to show this; details of the dose, frequency, and duration of use of vaginal oestrogens was not provided; and, it was not clear if tamoxifen and aromatase inhibitor subgroups were matched, as women on tamoxifen are inherently more likely to be lower risk [36–38]. International consensus guidelines continue to recommend women be offered vaginal oestrogens when other local therapies fail, after discussion regarding individual preferences and risks and benefits [15–18].

Only a small number of respondents had trialled vaginal laser (4%), which is unsurprising given difficulties with access due to cost and limited providers. The efficacy of vaginal laser in treating GUS remains uncertain. Evidence suggests a potential benefit in women with GUS [6, 39], including those with breast cancer [40, 41], however this is based largely on single arm trials. Safety concerns have also been raised with the FDA releasing a warning about vaginal laser [42] and Cruz et al. showing increased pain in women receiving laser alone compared to those using it in combination with a vaginal oestrogen [39]. Further evidence from randomised trials is needed to determine the safety and efficacy of this treatment option.

The strength of our study is the inclusion of a diverse group of respondents from multiple cancer centres and an online group with a range of ages and breast cancer stages and treatments. As with any survey, the perceptions of those completing the survey may not be representative of the broader population of people with breast cancer. It is possible that those with GUS were more likely to be interested in completing the survey. Additionally, the cross-sectional design of our survey did not allow exploration of how experiences or perceptions may change over time.

## Conclusions

Genitourinary symptoms are frequently reported by women with breast cancer, are frequently severe and can impact their quality of life. Despite this, most women in our study were not warned or asked about these symptoms. Routine assessment for GUS as a part of breast cancer follow-up is vital. Greater education and dissemination of information and resources with a focus on treatment options is needed to reach both health professionals treating women with breast cancer and women themselves. This will empower both groups to actively discuss these symptoms and provide treatment recommendations where appropriate.

## Data Availability

The datasets generated during and/or analysed during the current study are available from the corresponding author on reasonable request.

## Abbreviations

BCNA: Breast Cancer Network of Australia
DIVA: Day-to-day impact of vaginal ageing
BC: Breast Cancer
FDA: Food and Drug Administration
GUS: Genitourinary symptoms
ICIQ FLUTS: ICIQ Female lower urinary tract symptoms
